# M2 microglia-derived exosomes promote vascular remodeling in diabetic retinopathy

**DOI:** 10.1186/s12951-024-02330-w

**Published:** 2024-02-09

**Authors:** Xingxing Wang, Changlin Xu, Cunxin Bian, Pengfei Ge, Jie Lei, Jingfan Wang, Tianhao Xiao, Yuanyuan Fan, Qinyuan Gu, Hong-Ying Li, Jingyi Xu, Zizhong Hu, Ping Xie

**Affiliations:** Department of Ophthalmology, The First Affiliated Hospital of Nanjing Medical University, Nanjing, 210029 China

## Abstract

Diabetic retinopathy (DR) is a vision-threatening diabetic complication that is characterized by microvasculature impairment and immune dysfunction. The present study demonstrated that M2 microglia intensively participated in retinal microangiopathy in human diabetic proliferative membranes, mice retinas, retinas of mice with oxygen-induced retinopathy (OIR) mice, and retinas of streptozotocin-induced DR mice. Further in vivo and in vitro experiments showed that exosomes derived from M2 polarized microglia (M2-exo) could reduce pericyte apoptosis and promote endothelial cell proliferation, thereby promoting vascular remodeling and reducing vascular leakage from the diabetic retina. These effects were further enhanced by M2-exo that facilitated M2 polarization of retinal microglia. Collectively, the study demonstrated the capability of M2-exo to induce retinal microvascular remodeling, which may provide a new therapeutic strategy for the treatment of DR.

**Graphical Abstract:**

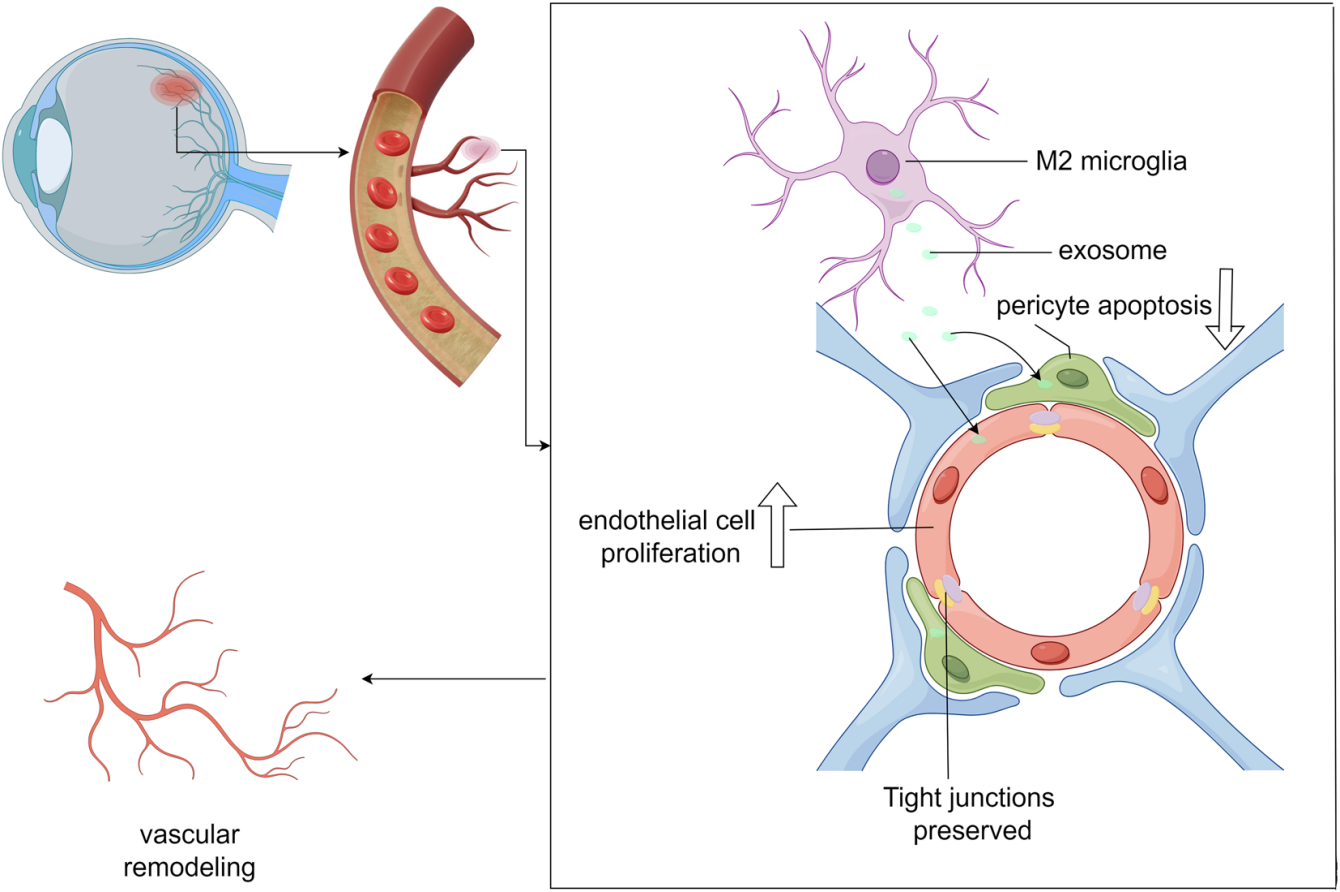

**Supplementary Information:**

The online version contains supplementary material available at 10.1186/s12951-024-02330-w.

## Introduction

Diabetic retinopathy (DR) is a highly prevalent condition, affecting 34.6% of the estimated 500 million people with diabetes mellitus worldwide. Among these, approximately 30% of people have been reported to have varying degrees of visual impairment [1]. DR is a diabetic microvascular complication [2] accompanied by morphological changes such as thickening of basement membrane and damage of tight junctions in endothelial cells (ECs) as well as loss of pericytes, which leads to increased vascular permeability or impaired vascular function [3]. Reportedly, the EC-to-pericyte ratio is an essential factor for the formation and maintenance of functional vessels during angiogenesis, vascular maturation, and stabilization [4]. Therefore, the prevention of pericyte loss and regulation of dysfunctional EC in early DR can help avoid vascular abnormalities in DR.

Accumulating evidence indicates that the immune system plays an important role in the progression of DR [2, 5, 6]. Microglia are important intrinsic immune cells in the retina, are usually located in the inner retina, and help monitor and clear retinal metabolites [7]. It is well known that activated microglia can be classified into two phenotypes: M1 inflammatory microglia and M2 anti-inflammatory microglia [8]. In the early stages of DR, microglia shift from the surveillance state M0 to the activated state M2, specifically to neutralize harmful stimuli and restore tissue homeostasis [9]. However, prolonged progression of the DR leads to the transformation of microglia to the secretory M1 phenotype, which secretes pro-inflammatory mediators, including cytokines and chemokines [7]. Reportedly, M1 cells produce large amounts of oxidative metabolites and pro-inflammatory cytokines that counteract invading pathogens and tumor cells. In contrast, M2 cells promote angiogenesis and remodel the stroma to orchestrate homeostasis in vivo following inflammatory responses, which have been associated with the regression of chronic leg ulcers [10], atherosclerotic lesions [11], and traumatic spinal cord injuries [12]. Recently, studies have demonstrated the involvement of M2 microglia in retinal neovascularization in patients with DR as well as in mice with oxygen-induced retinopathy (OIR) and with streptozotocin (STZ)-induced DR [13–15].

However, limited studies have assessed the mechanism by which M2 microglia interact with retinal neovascularization. Exosomes are lipid-layered vesicles belonging to a class of extracellular carriers (30–150 nm in diameter) that are secreted by cells. These can form complexes with peripheral or distant cells in body fluids to mediate signaling and thus participate in various physiological or pathological processes such as immune regulation, cell differentiation, and vascular neogenesis [16]. During the past decade, several lines of evidence have demonstrated the important roles of exosomes in intercellular communication and cell signaling. However, the role of exosomes in diabetes mellitus-induced vascular dysfunction is still largely unknown.

Accordingly, the current study explored whether M2-microglia derived exosomes (M2-exo) promote vascular remodeling by regulating the EC-to-pericyte ratio, thereby reversing microvascular impairment caused by DR.

## Materials and methods

### Patients and tissue samples

To investigate the role of M2 microglia in DR, 20 proliferative membranes were collected from 20 patients with proliferative diabetic retinopathy (PDR) and 15 from 15 patients with proliferative vitreoretinopathy (PVR) (as controls). None of the patients were treated with anti-VEGF medications. All proliferative membranes were collected intraoperatively. The patients were enrolled from 2019 to 2020 at the First Affiliated Hospital of Nanjing Medical University (Additional file 3: Table S1).

This study was conducted in accordance with the Declaration of Helsinki and was approved by The Ethics Committee of the Faculty of Medicine, Nanjing Medical University (2017-SR-283). All patients provided informed consent to participate in the study.

### Cell culture

Human primary endothelial cells (hRECs) used in the experiments were purchased from the American Type Culture Collection. Two to three generations of hRECs were used. Primary cultures of mouse microglia were obtained according to an established protocol with minor modifications, and their purity was verified using flow cytometry [17]. Subsequently, primary extraction and culture of pericytes from mouse retina were performed based on a previous protocol, and the purity of cultured pericytes was verified by immunofluorescence [18]. HRECs were cultured in endothelial cell medium (5.5 or 30 mM glucose), and pericytes were cultured in pericyte medium (5.5 or 30 mM glucose). To investigate the effect of exosomes from M2 microglia on DR, hRECs and pericytes were cultured in a medium containing normal glucose (5.5 mM) or high glucose (30 mM), as an osmotic pressure control.

### Exosome isolation and identification

The extraction of exosomes from the culture medium supernatant of microglia with and without IL-4 pretreatment. Briefly, microglial cells were cultured with and without IL-4 for 48 h. After incubation, the microglia were washed twice with phosphate-buffered saline (PBS). The PBS was replaced with an exosome-free medium and then the medium was collected for further analysis. Subsequently, the collected medium was centrifuged at 300×*g* for 10 min and then at 2000×*g* for 10 min. The cell supernatant obtained was filtered through a 0.22-μm filter to remove cellular debris and then transferred to an Amicon Ultra-15 centrifuge filter (Millipore, USA) and centrifuged at 4000×*g* until the volume of the upper chamber was reduced to approximately 200 μL. To purify the exosomes, the liquid from the upper chamber was loaded on 30% sucrose/D2O pads and then subjected to ultracentrifugation at 100,000 g (Beckman Coulter) for 60 min at 4 °C. The pellet obtained was suspended in PBS for further analysis.

The exosomes obtained were morphologically characterized using transmission electron microscopy (TEM; Tecnai 12; Philips, Best, The Netherlands). The diameter and number of exosomes were determined using nanoparticle tracking analysis (NTA, Nanosight Ltd., Navato, CA). Western blot analysis was performed to examine the surface biomarkers of exosomes.

### Uptake of exosomes by hRECs and pericytes

Briefly, 4 mg/mL of Dil solution (Molecular probe, USA) was incubated with PBS-containing exosomes for fluorescent labeling. Excess dye was removed by centrifugation at 100,000×*g* at 4 °C, and the labeled exosomes were washed thrice with PBS. These Dil-labeled exosomes were then co-cultured with hRECs and pericytes for 37 °C 24 h. Subsequently, the cells were washed with PBS and fixed in 4% paraformaldehyde for 15 min. The uptake of exosomes by cells was observed using laser confocal microscopy (Carl Zeiss Microscopy GmbH, Germany) (Additional file 1: Video S1).

### Cell proliferation assay

Cell proliferation was assessed using the EdU Cell Proliferation Kit and the Alexa Fluor 596 Imaging Kit (Thermo Fisher Scientific), according to the manufacturer’s instructions. Briefly, hRECs in normal (5.5 mM glucose) or high-glucose medium (30 mM glucose) were seeded into 96-well plates at an initial density of 5 × 10^3^ cells/well and were co-cultured with 100 mg/mL M0-exo or M2-exo for 24 h. Subsequently, 50 mM EdU medium was added to each well and incubated for 2 h at 37 °C. The cells were then washed twice with PBS for 10 min each. They were then fixed with 4% paraformaldehyde (PFA) for 15 min, neutralized with 2 mg/mL glycine, and then washed with PBS before permeabilization with 0.5% Triton X-100 for 10 min. Finally, the hRECs obtained were labeled with Apollo-596 stain; excess stain was removed by washing thrice with 0.5% Triton X-100. EdU assays were performed three times independently, and a total of three areas were randomly selected for confocal fluorescence microscopy imaging under both glucose conditions (MIC00223 LSM5 Live). The percentage of EdU-positive cells (red markers) was calculated and analyzed using the ImageJ software.

### Transwell assay

The Transwell assay was conducted using a 24-well chamber containing a membrane filter insert. After 48 h of treatment, each set of transpore chambers was removed, and the cells on the surface of the apical chamber were removed using a cotton swab. Only the cells migrating to the lower side of the membrane were collected and fixed with 4% PFA for 15 min, followed by staining with 0.5% crystal violet solution for 15 min and washing thrice with PBS.

The experiments were repeated thrice independently. A total of three areas were randomly selected for imaging under each condition. Micrographs were obtained using a DP71 digital camera (Olympus).

### Tube formation assay

Briefly, 96-well plates were coated with 50 µL of basement membrane matrix (Matrigel; BD Biosciences, USA), followed by seeding hRECs at a density of 1 × 10^6^ cells/well. The cells were then treated with endothelial cell medium for 8 h at 37 °C. The capillary structures formed by hRECs on Matrigel were photographed using a DP71 digital camera (Olympus) and were analyzed using the ImageJ software.

Tube formation assay was performed thrice independently, and a total of three areas were randomly selected for imaging under each condition.

### Blood–retinal barrier permeability of hRECs and pericytes in co-culture model

Briefly, pericytes were seeded in inserts (Corning, USA) with a pore size of 0.4 μm and cultured until confluence. hRECs were then spread evenly over the upper layer of pericytes and cultured until confluence. The culture medium was changed every alternate day. After the cells attained confluence and reached the upper chamber, PBS, M0-exo, or M2-exo was added to the upper chambers and allowed to incubate for 48 h. Then, FITC-dextran (70 kDa, 0.5 mg/mL) was added to the upper chamber for 1 h. Subsequently, the medium was collected from the bottom chamber and the fluorescence intensity of FITC-dextran was measured at 538 nm (Fig. 6E).

### Transepithelial electrical resistance (TEER) measurement

TEER was assessed to evaluate the barrier function between cells. Briefly, the cells were pre-equilibrated with Hank’s balanced salt solution for 30 min, followed by measurement of TEER using Millicell® ERS (Millipore, USA). TEER values were calculated using the following formula:

$${\text{TEER}}\;\left( {\Omega \cdot\;{\text{cm}}^{2} } \right) = \left[ {{\text{TEER}}\;{\text{total}} - {\text{TEER}}\;{\text{blank}}} \right] \times {\text{membrane}}\;{\text{area}}.$$

### Immunofluorescence assay

Cell or tissue sections were fixed with 4% polyoxyethylene and permeabilized with 0.3% Triton X-100, then blocked with 5% bovine serum albumin (BSA) and finally incubated overnight at 4 °C with the corresponding primary antibody for 24 h. Then, the sections were treated with corresponding secondary antibodies and DAPI reagents to assess immunoreactivity using fluorescence microscopy.

### Western blot assay

M0-exo and M2-exo concentrations were determined by BSA (Beyotime, China) according to the manufacturer’s instructions. Proteins from cells or tissues were extracted by RIPA lysis (Beyotime, China). Then, the proteins were denatured, followed by separation on SDS-PAGE gels. Subsequently, the proteins were transferred to PVDF membranes, which were then blocked using a blocking buffer (Beyotime, China) for 1 h at room temperature, followed by overnight incubation at 4 °C with the primary antibody. After washing with Tris-buffered saline with 0.1% Tween® 20 detergent, the membrane was incubated with the corresponding secondary antibody for 2 h. The protein bands obtained were visualized using a PowerOpti-ECL detection system (ThermoFisher Scientific, USA).

### In vivo EdU proliferation assay

EdU (50 μg per day at a concentration of 125 μg EdU per 100 μL PBS) was intraperitoneally administered to mice with OIR on 5 consecutive days from p17 to p21. Mice were sacrificed at p21. Eye fixation and flat-mount preparation were performed. Retinal was stained by the EdU Kit (Beyotime, China) according to the manufacturer’s protocol.

### Flow cytometry

Eyecups of PBS-perfused mice were dissected to separate the retina from adjacent tissue. Subsequently, the retinal tissue was dissociated by resuspension, and dead cells were excluded by incubating in the fixable viability dye 450 (Thermo Fisher Scientific). Cells were stained with anti-CD45, anti-CD11b, anti-F480, and CD206 (1:200; Thermo Fisher Scientific) at 4 °C for 30 min. Then, the cells were washed and analyzed using CytoFlEX (Beckman Coulter, USA) to sort retinal cells. Flow results data were obtained using the CytExpert software (Beckman Coulter, USA).

Apoptosis was examined by flow cytometry analysis. For apoptosis analysis, cells were collected, washed with PBS, and incubated with Annexin V-FITC/PI Apoptosis Detection Kit (Vazyme) for 15 min. Apoptosis was then analyzed by flow cytometry CytoFlEX (Beckman coulter, USA) according to the manufacturer’s instructions. The CytExpert software (Beckman coulter, USA) was used to analyze flow cytometry data. Results are expressed as means ± standard deviation of three independent experiments.

### Establishment of the OIR mouse model

All the animal experiments were approved by the Committee on the Ethics of Animal Experiments of Nanjing Medical University.

OIR mouse model has been widely used for the mechanism research of retinal proliferative microvascular disease [19, 20]. Mice and their lactating dams were housed in an oxygen supply chamber (oxygen concentration 75% ± 3%) from p7 to p12 and were subsequently returned to room air, as previously reported [21]. All mice were housed and maintained at a constant temperature with a 12-h light/dark cycle. The weight of each pup was closely monitored to determine adequate metabolic health. Mice in the control group were housed in room air and sacrificed at their specified time points.

### Establishment of STZ-induced DR mouse model

After 2 weeks of adaptive feeding, 30 C57BL/6 mice (weight 18–22 g) were intraperitoneally administered 55 mg/kg STZ for 5 consecutive days. In contrast, the other 30 mice were treated with normal, while the other 16 mice were treated with normal saline and served as the control group. Serum glucose concentrations were monitored 7 days after treatment initiation, and mice with glucose levels > 16.7 mmol/L were considered diabetic. Mice with diabetes received an intravitreal injection of either PBS (n = 10), M0-exo (n = 10), or M2-exo (n = 10). In the STZ group, six mice from each group were administered Evans Blue (2% saline, 200 μL; tail vein injection) for disrupting the blood–retinal barrier (BRB) (Sigma) 3 months after STZ injection. After 3 h, mice were sacrificed and their retinas were removed and homogenized in a trichloroacetic acid and ethanol solution (1:2). The homogenized tissue was then incubated at 60 °C for 24 h, followed by centrifugation at 10,000×*g* for 10 min. The supernatant obtained was collected, and fluorescence was quantified using a spectrophotometer at an excitation wavelength of 620 nm and an emission wavelength of 680 nm (Fig. 4A).

### Trypsin-digested vessel preparation

At autopsy, each eye was removed and then fixed in 4% paraformaldehyde for 24 h. The retinas were isolated under a dissecting microscope and were then washed completely with copious amounts of PBS. The retinas were then incubated in 3% trypsin in sodium phosphate buffer for 1 h. After completion of digestion, the isolated retinal vessels were stained with periodic acid-Schiff hematoxylin (PAS) and were visualized for.

### Statistical analysis

Statistical analyses were performed using GraphPad Prism 8.0 (GraphPad Software Inc., USA). Student’s t-test was used for the comparison of data between two groups. One-way or two-way analysis of variance tests were used for multivariate analysis. Data were expressed as means ± standard deviations. Results with a P-value < 0.05 were considered significant.

## Results

### M2 microglia mediate physiological and pathological retinal angiogenesis

**Fig. 1:**
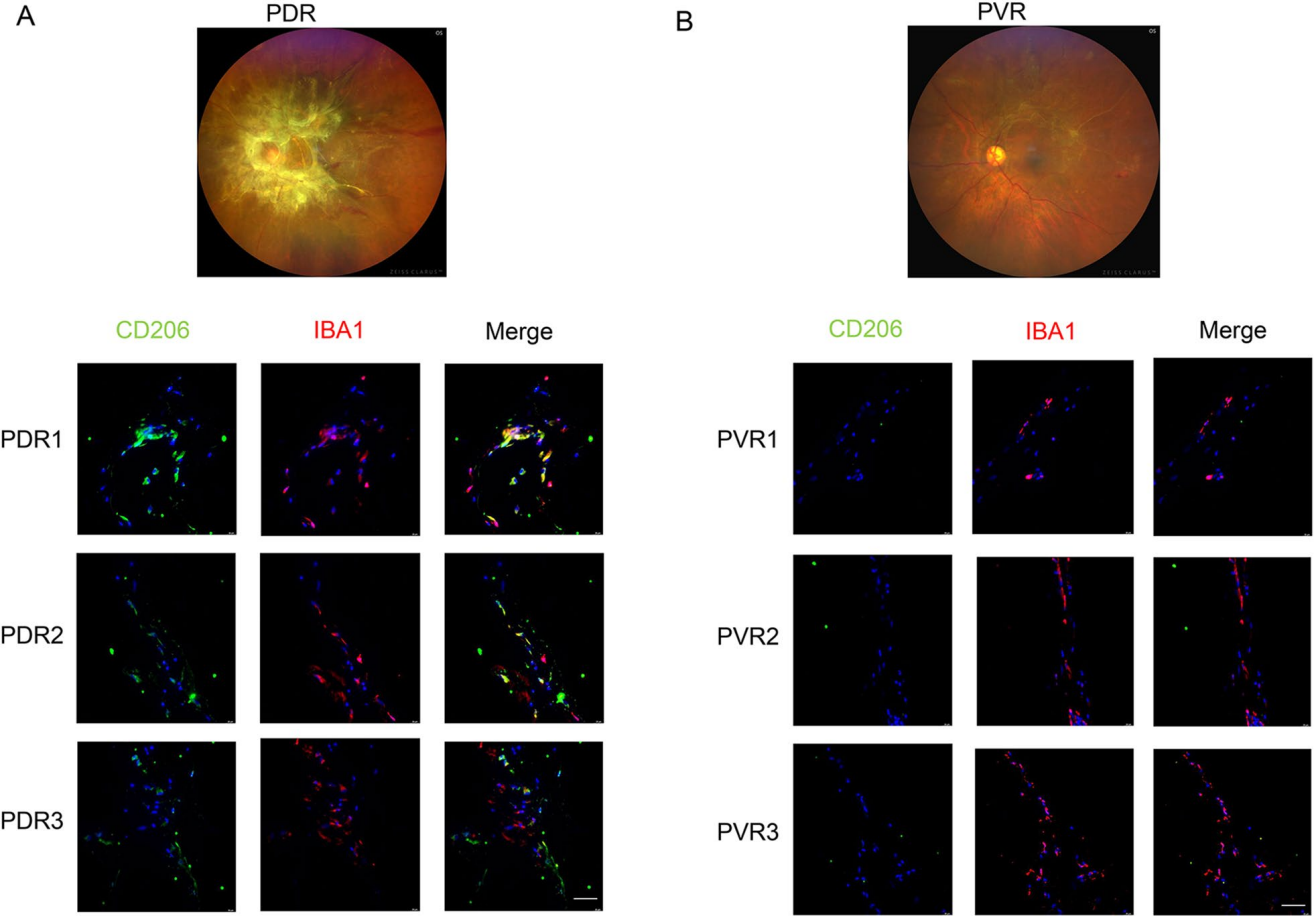
Immunohistochemical staining of M2 microglia in the epiretinal fibrovascular membrane obtained from patients with PDR (**A**) and PVR (**B**) (scale bar = 50 μm)

**Fig. 2:**
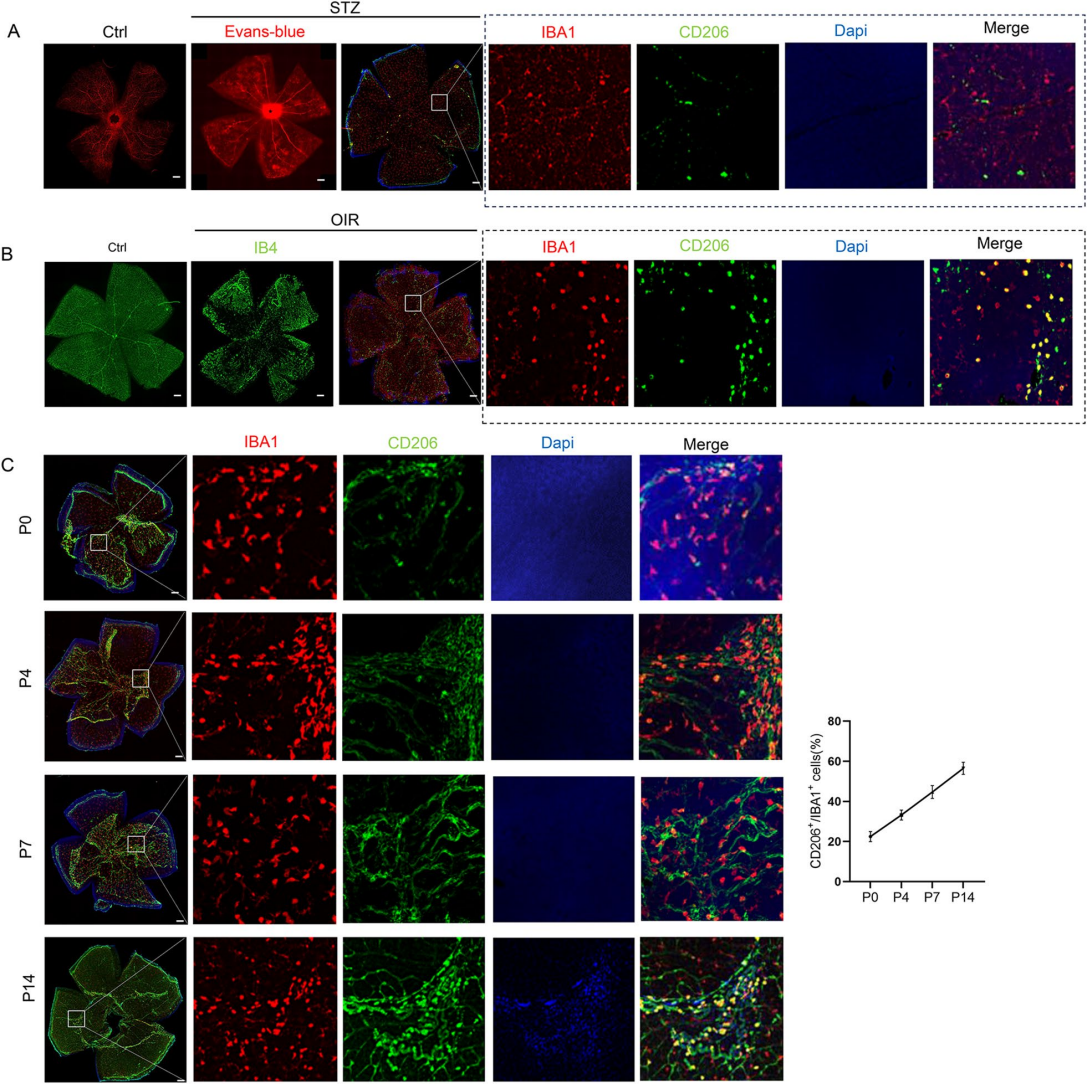
Role of M2 microglia in OIR and STZ-induced DR mouse models (scale bar = 200 μm). **A** Involvement of M2 microglia in retinal vascular changes in STZ-induced DR mouse model. **B** Involvement of M2 microglia in retinal vascular changes in OIR mouse model. **C** Involvement of M2 microglia in normal blood vessel formation

To verify whether M2 microglia were activated under conditions of DR, we first performed immunofluorescence staining of surgically harvested proliferating membranes from patients with PDR and PVR. CD206, a classic marker of M2 microglia, was significantly elevated in the fibrovascular membranes of patients with PDR than in those of patients with PVR (Fig. 1A, B). Microglia were labelled with Iba1 antibody and retinal vascular endothelial cells with Isolectin B4 (IB4). Evans blue leakage was evident in STZ-induced retinas of DR mice. Co-staining of IBA1 and CD206 confirmed the involvement of M2 microglia in the retina of DR mice (Fig. 2A). At P17, the co-staining of IBA1 and CD206 confirmed the involvement of M2 microglia in the retina with OIR (Fig. 2B). Furthermore, we assessed M2 microglia activation in physiologically developed retinas. We found that the retinas of mice at postnatal days 0, 4, 7, and 14 demonstrated persistent activation of M2 microglia, suggesting that M2 microglia may participate in physiological angiogenesis as well (Fig. 2C).

### M2-exo promoted retinal vascular remodeling in OIR mice

**Fig. 3:**
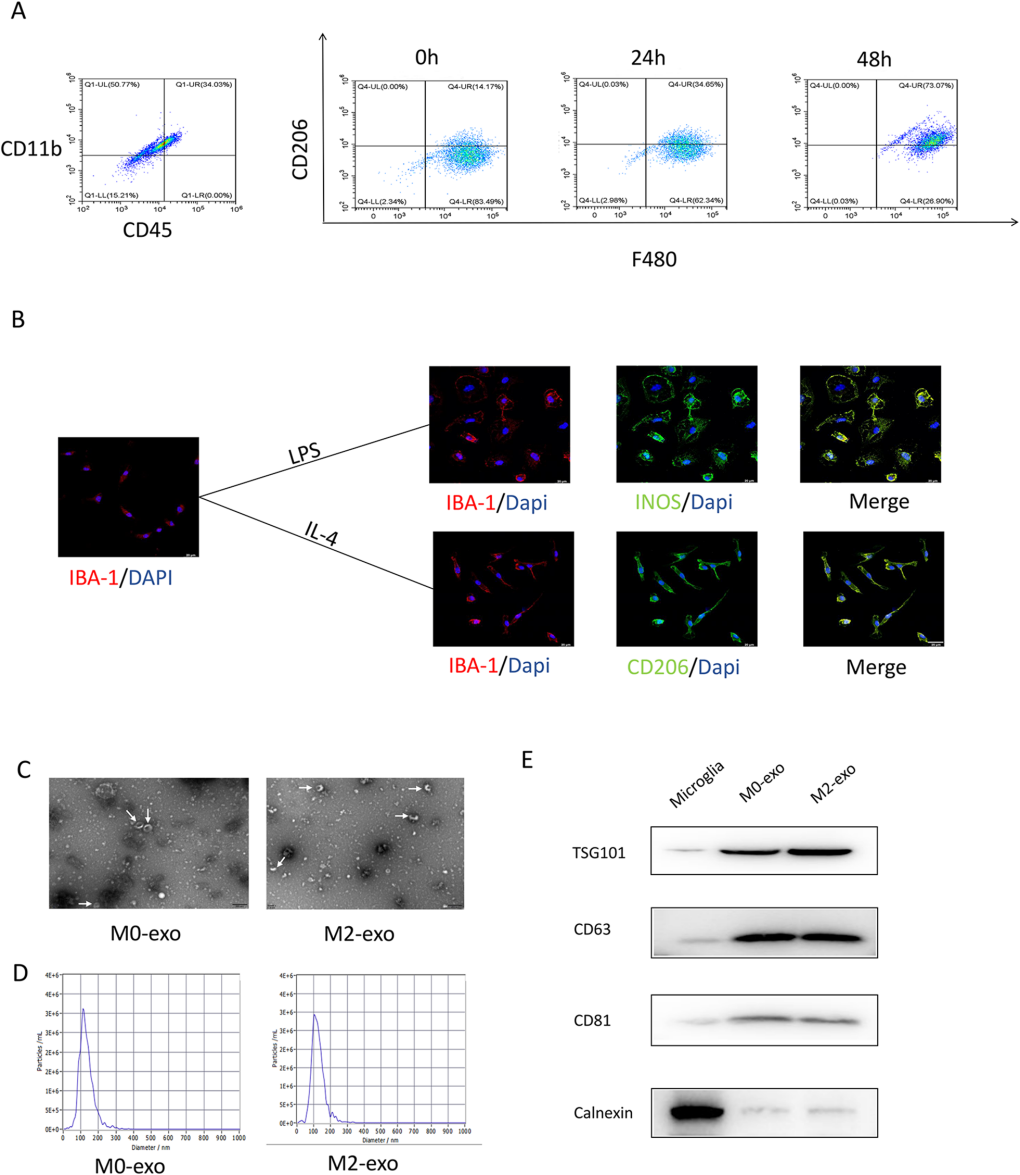
Identification of M2 microglia and exosomes. **A** Microglia were induced with IL-4 for 0, 24, and 48 h for flow identification. **B** Identification of primary microglial polarization by immunofluorescence (scale bar = 20 μm). **C** TEM images of exosome ultrastructure (white arrows represent exosomes). **D** Nanoparticle tracking analysis of M0 and M2 exosomes. **E** Representative bands showing protein levels of the exosomal surface markers TSG101, CD63, CD81, and calnexin

To investigate if M2 microglia-derived exosomes are involved in retinal angiogenesis, primary microglia were extracted and then stimulated with lipopolysaccharide (LPS) or IL-4 to induce M1 or M2 polarization, respectively. The purity of the primary microglia obtained was assessed by flow cytometry. The results revealed that cells with CD11b+/CD45-positive microglia accounted for > 85% of the cells tested. After being stimulated with IL-4 for 0, 24, and 48 h, CD206 expression was the highest at 48 h (Fig. 3A). Immunofluorescence staining further verified that primary microglia stimulated with LPS (1 μg/mL) for 48 h increased the expression of iNOS, a marker of M1 microglia. In contrast, treatment of microglia with IL-4 (20 ng/mL) for 48 h resulted in increased expression of CD206 (Fig. 3B).

Next, IL-4-treated or untreated microglia were subjected to ultracentrifugation to extract M2-exo or M0-exo (as controls). The morphology and size distribution of exosomes were then verified by TEM and NTA (Fig. 3C, D). The typical cup-shaped morphology of membrane vesicles was observed, and the diameter of membrane vesicles was in the range of 30–150 nm in both M2-exo and M0-exo. Western blot analysis showed that exosome markers, such as CD63, CD81, and TSG101, were expressed in exosomes but not in the negative control cells. In contrast, Calnexin was mainly detected in cells than in exosomes (Fig. 3E).

**Fig. 4:**
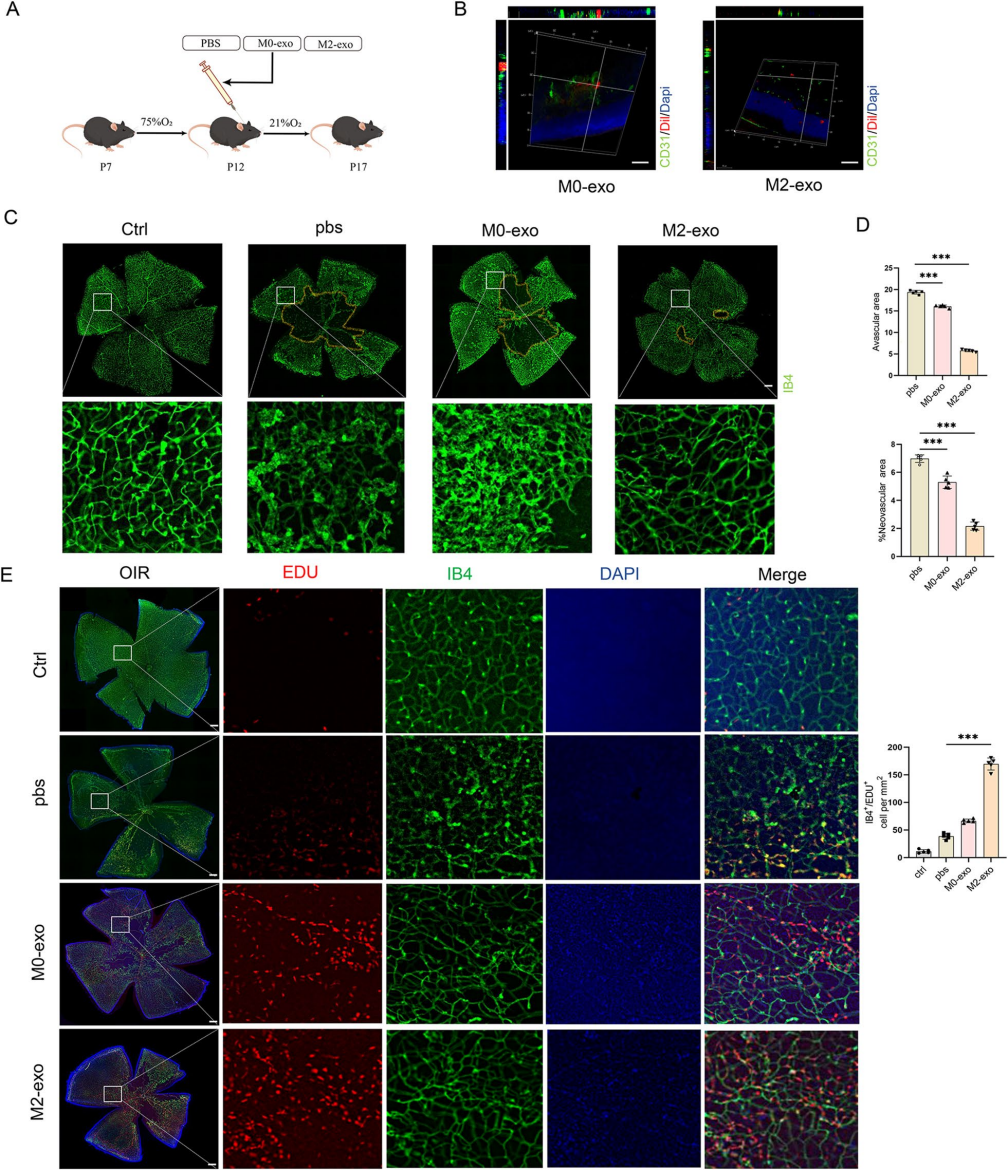
Promotion of in vivo retinal angiogenesis and remodeling by exosomes of M2 microglia. **A** Flow chart describes the in vivo establishment of the OIR mouse model. **B** Three-dimensional view of a frozen retinal section of OIR mouse showing exosome uptake in vivo. Scale bar = 50 μm. **C**, **D** Retinal flat mounts of C57BL/6J mice after OIR induction at P17 and intravitreal injection of M0-exo, M2-exo, or PBS at P12; yellow indicates the avascular area. Compared with the PBS or M0-exo group, the M2-exo group demonstrated significantly reduced avascular region and abnormal proliferating vessels. *p < 0.05, **p < 0.01***p < 0.001; n = 5. Scale bar = 200 μm. **E** Retinal flat mounts after tail vein injection of EdU in OIR mice. Effects of M2-exo on cell proliferation are demonstrated as the highest EdU+ cell number. ***p < 0.001; n = 5. Scale bar = 200 μm

OIR mice at P12 were intravitreally administered PBS, M0-exo, or M2-exo. Immunofluorescence analysis of mice retinas injected with Dil-labeled exosomes at P12 days demonstrated co-staining with Dil and CD31, a marker of vascular endothelial cells, which indicated the uptake of exosomes by vascular endothelial cells (Fig. 4B). To evaluate the effect of M2-exo on angiogenesis, the OIR model P17 retinas were further made flat-mount and stained with IB4.Retinal flat-mount images showed a typical central avascular area in OIR mice. However, significant retinal remodeling was observed in the M2-exo group, as evidenced by a reduction of the area of pathological neovascularization and the avascular zone (Fig. 4C, D). To further determine that M2-exo promotes vascular endothelial proliferation, we did in vivo EDU in OIR model mice. Furthermore, OIR and control mice at P12 were injected with EdU for 5 consecutive days. The number of EdU+ cells/mm^2^ increased after M2-exo treatment compared with blank control (OIR-blank), vehicle control (OIR-PBS), or M0-exo treated groups (Fig. 4E).

### M2-exo protected BRB by normalizing endothelial cell-to-pericyte ratio

**Fig. 5:**
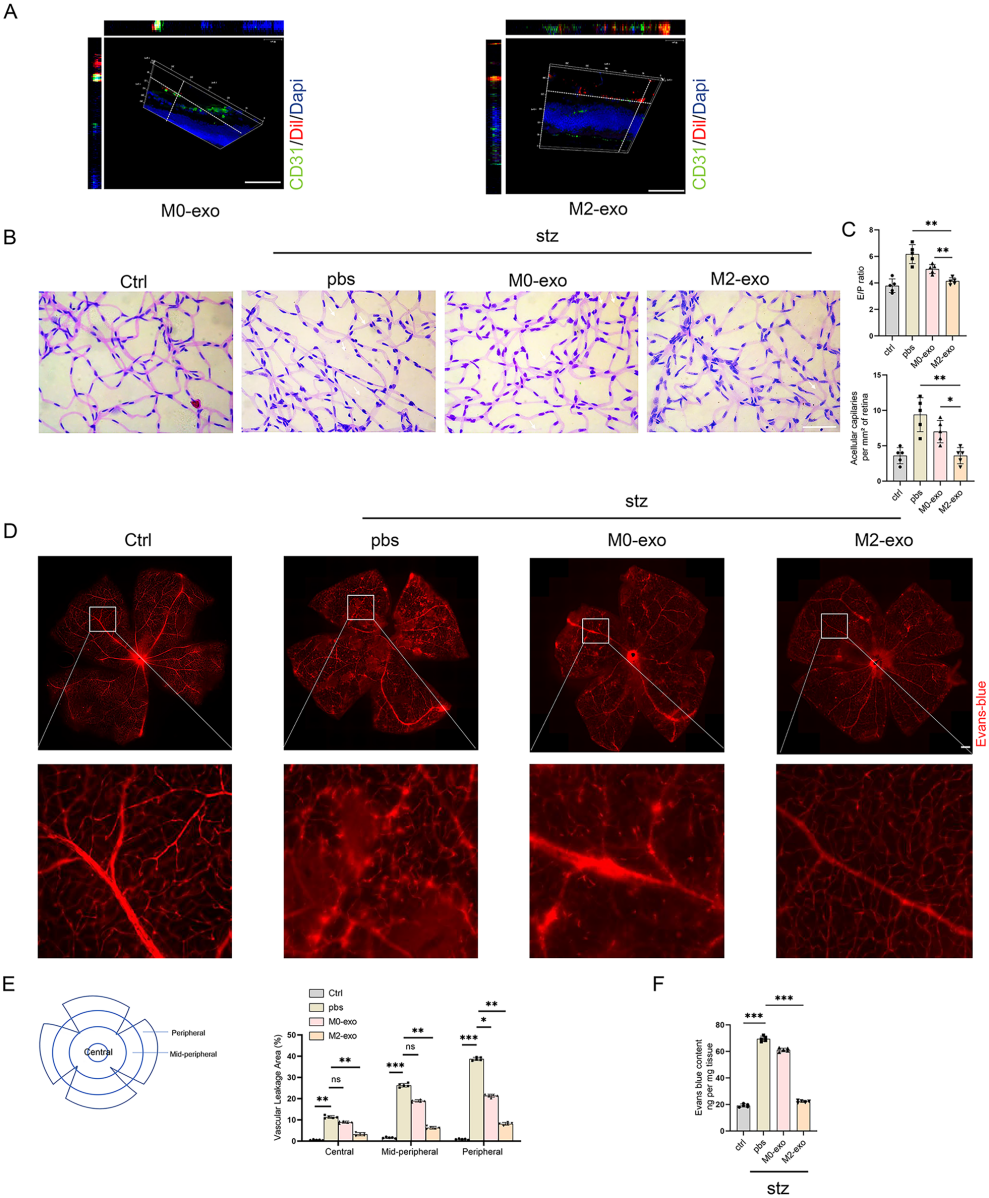
M2-exo attenuates retinal vascular leakage and reduces the number of acellular capillaries. **A** Three-dimensional view of a frozen retinal section of mice with STZ-induced DR showing exosome uptake in vivo. Scale bar = 100 μm. **B**, **C** E/P ratio, number of acellular strands, and morphological changes in vessels among the four groups assessed using PAS staining of retinal tissue (*p < 0.05, **p < 0.01). White arrows indicate acellular strands. n = 5. Scale bar = 50 μm. *E/P* endothelial cell-to-pericyte. **D**, **E** Microvascular permeability of whole retinal branches evaluated by Evans blue fluorescence staining. Dye leakage was observed outwards from the retinal vessels in the STZ-induced DR mouse model, whereas almost no Evans blue leakage was observed in the M2-exo-treated group. *p < 0.05, **p < 0.01***p < 0.001; n = 5. Scale bar = 200 μm. **F** Quantification of Evans blue content in the retina. ***p < 0.001; n = 5

Recent evidence has demonstrated that the interactions between endothelial cells and pericytes in blood vessels play a central role in regulating vascular formation, stabilization, remodeling, and function. To further determine the role of M2-exo in DR, we next investigated its effects on STZ-induced DR mice. PAS staining of the retinal vascular network revealed that the vascular endothelial nuclei were long oval or irregular and that the pericyte nuclei were round or triangular and dark. Moreover, STZ-induced diabetic mice sections co-stained of Dil (labeling exosomes) and CD31 was observed, which indicated the uptake of exosomes by vascular endothelial cells (Fig. 5A). After injecting PBS or exosomes, the endothelial cell-to-pericyte ratio and the number of acellular strands were higher in the retinas of mice with STZ-induced DR (PBS group) but were attenuated in the M2-exo group (Fig. 5B, C). Meanwhile, vascular leakage, assessed using Evans blue fluorescence staining, was increased in mice with STZ-induced DR. In addition, the vascular network was disorganized and characterized by multiple dotted and lamellar hyperfluorescent leakages. However, M2-exo treatment significantly reduced retinal vascular leakage, increased the clarity of vascular texture, and significantly reduced the localized lamellar hyperfluorescence shadow (Fig. 5D, E). In addition, the leakage in the M2 exosome group was significantly reduced, as demonstrated by Evans blue staining of retinal tissues (Fig. 5F). These experiments suggest that M2-exo treatment can alleviate retinal microangiopathy in mice with DR.

### Protective effect of M2-exo on retinal vascular endothelial cell and pericyte

**Fig. 6:**
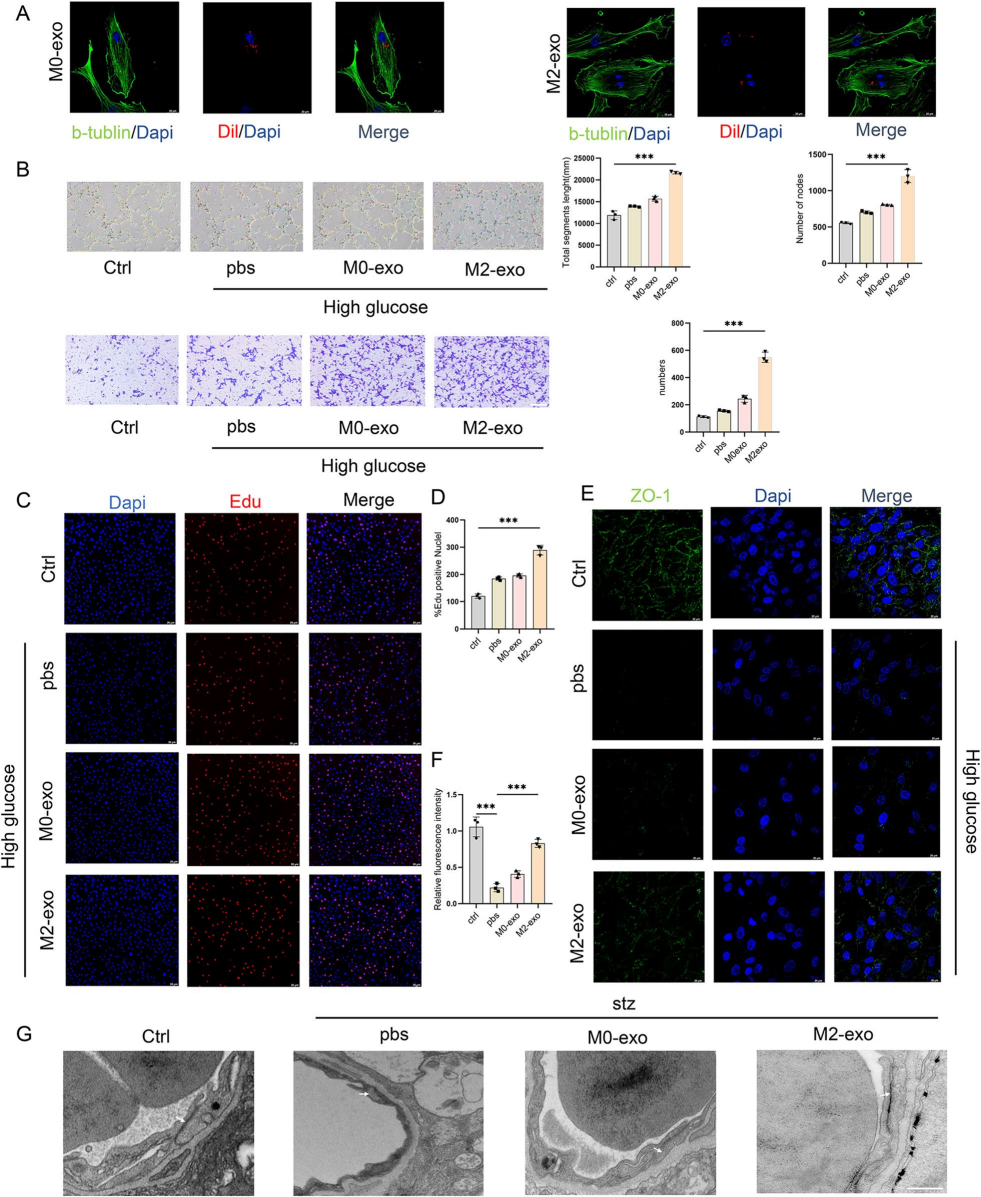
Effect of exosomes from M2 microglia on hRECs. **A** The uptake of hRECs into exosomes was detected by labeling them with Dil. Scale bar = 20 μm. **B** Tube formation ability was measured. Migration of hRECs was determined by Transwell assay. Scale bar = 200 μm. **C**, **D** Proliferation of hRECs was assessed by the EdU assay. The percentage of EdU-positive cells (marked in red) was calculated using three randomly selected regions. Scale bar = 20 μm. **E**, **F** Immunofluorescence detection and quantification of ZO-1 in hRECs. Scale bar = 20 μm. **G** The morphology of vascular TJs in each group of mice was observed by TEM (arrows represent TJs). Scale bar = 2 μm

To further investigate the effects of M2-exo on the vascular endothelium and pericytes, we next explore its effects on endothelial cells and pericytes in vitro. In in-vitro studies, we first demonstrated that M0-exo and M2-exo, incubated with Dil, were taken up by hRECs (Fig. 6A). Compared with the groups of blank control (normal glucose) and high glucose with PBS or M0-exo, M2-exo showed enhanced tube-forming ability and migration of hRECs (p < 0.001, Fig. 6B). In addition, compared with blank control, PBS, or M0-exo treated groups, M2-exo group demonstrated increased proliferation of hRECs, as observed using the EdU assay (p < 0.001, Fig. 6C, D). In addition, immunofluorescence staining confirmed that ZO-1 expression was significantly increased after M2-exo treatment (Fig. 6E, F). Tight junctions (TJs) are apical intercellular junctions that maintain the physiological barrier between adjacent endothelial cells [22]. In the present study, TJs were preserved in mice with STZ-induced DR following M2-exo treatment, as assessed by TEM (Fig. 6G).

**Fig. 7:**
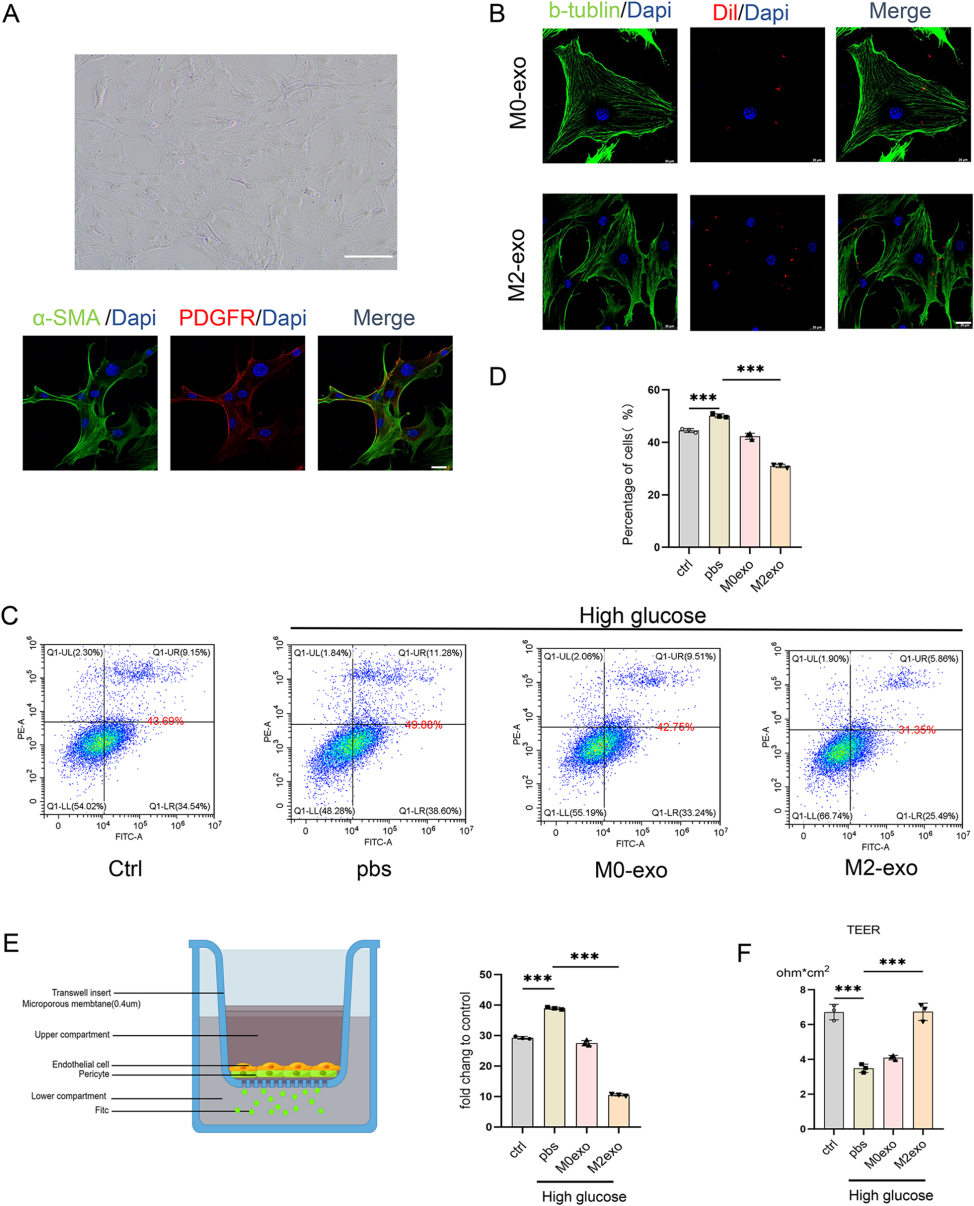
Effect of M2 microglia-derived exosomes on pericytes. **A** Identification of pericyte fluorescent staining (scale bar = 20 μm) and pericyte morphology using light microscopy. Scale bar = 200 μm. **B** Uptake of M2 exosomes by pericytes. Scale bar = 20μm. **C**, **D** Flow cytometry analysis of pericytes for apoptosis and quantification. **E**, **F** Blood–retinal barrier integrity was assessed by transendothelial electrical resistance (TEER) and fluorescein isothiocyanate (FITC)-dextran in co-cultures of hRECs with pericytes

Early DR is also associated with a loss of pericytes, which leads to the destruction of the BRB [23]. In this study, we extracted peripapillary retinal cells with a purity of > 85% (Fig. 7A). After M0-exo and M2-exo were taken up by pericytes (Fig. 7B), flow cytometry analysis showed that M2-exo could significantly reduce pericyte apoptosis under high-glucose conditions (Fig. 7C, D). Next, we evaluated the effect of M2-exo on vascular endothelial cell–pericyte barrier function. As shown in Fig. 7E, pericytes were first seeded at the bottom of the upper chamber, covered by a layer of hRECs, and were then co-cultured with normal- or high-glucose media. As expected, adding M2-exo significantly increased the TEER value of pericytes (Fig. 7F) and decreased the permeability of FITC-dextran (Fig. 7E).

### M2-exo further promoted microglia M2 polarization in the retina

**Fig. 8:**
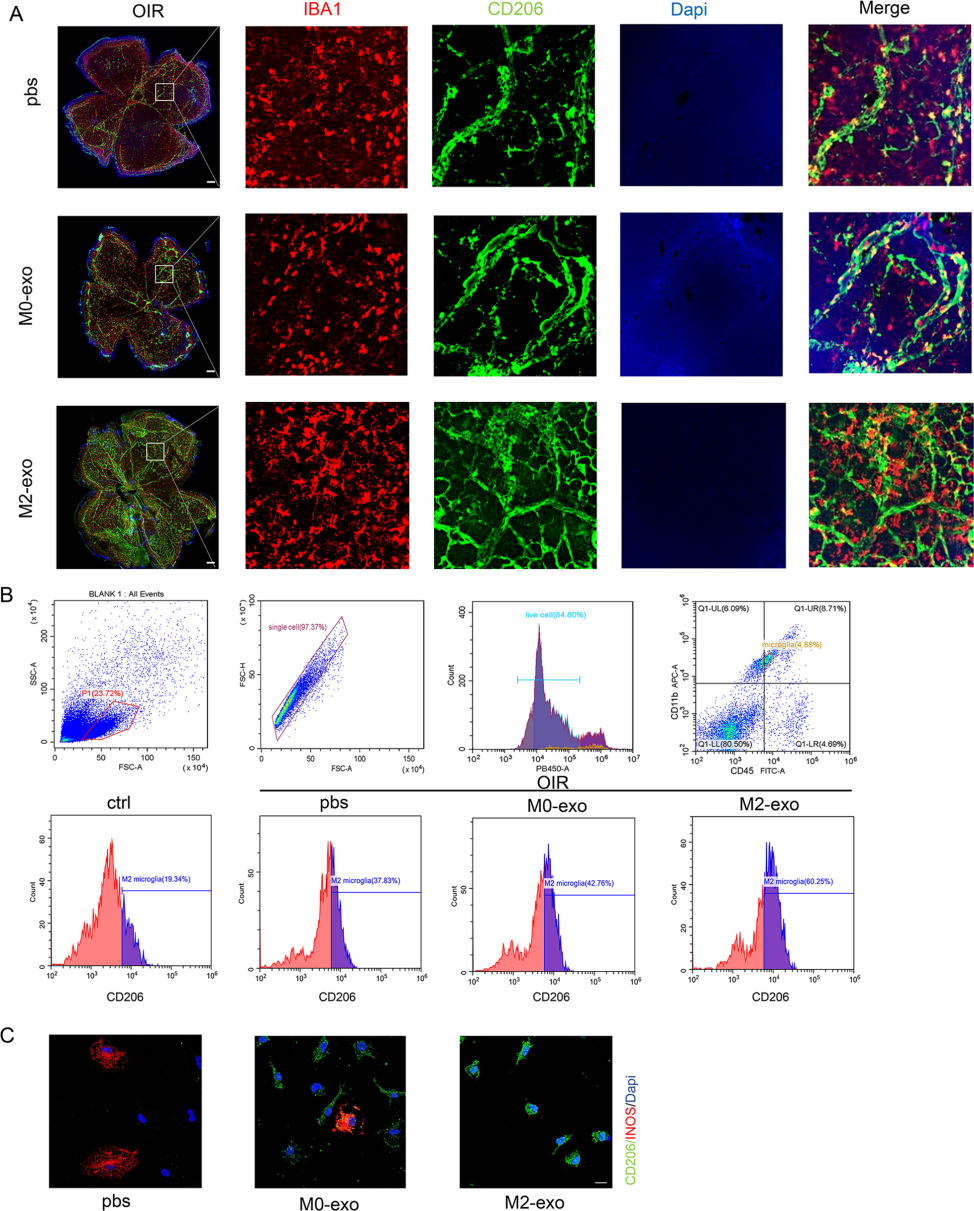
M2-exo promotes revascularization and facilitates M2 polarization of microglia. **A** Validation of the ability of M2-exo to promote revascularization and M2 microglia polarization by immunofluorescence in OIR mouse (scale bar = 200 μm). **B** Retinal cell flow analysis demonstrates that M2-exo promotes M2-type microglial polarization. **C** Representative immunofluorescence images showing changes in primary microglia after treatment with PBS, M0-exo, and M2-exo. Scale bar = 20 μm

Interestingly, M2-exo could further polarize microglia toward M2 in the retinas of OIR mice at P12 (Fig. 8A). We found that M2-exo promotes retinal microglia polarisation towards M2 in both OIR model and STZ model mice Additional file 2: Fig. S1. In addition, flow cytometry analysis of the retinas, following digestion into single-cell suspensions, verified that M2-exo treatment increased the proportion of M2 polarized microglia (Fig. 8B). Meanwhile, among PBS, M0-exo, and M2-exo treatments, only M2-exo treatment induced the polarization of primary microglia towards the M2 type (Fig. 8C). These results suggested that M2-exo might promote microglia M2 polarization, thus upregulating the amount and effect of M2-exo in facilitating retinal vascular remodeling.

## Discussion

Exosomes have recently gained attention because of their roles in intercellular communication and diseases. In this study, we investigated the role of M2-exo in maintaining vascular endothelial function. The results revealed that M2-exo could regulate microvascular dysfunction in mice with OIR and STZ-induced DR. Mechanistically, M2-exo normalizes the ratio of endothelial cells/pericytes, thereby protecting the BRB.

DR is a chronic, progressive eye disease that often results in functional and structural damage to the retina and is characterized by retinal cell apoptosis, thickening of the retinal vascular basement membrane, disruption of BRB, and abnormal neovascularization [24]. A recent pathological study of retinal tissues from patients with diabetes revealed that pericyte loss might be the initial event following BRB disruption [25]. The loss of retinal pericyte often changes the retinal endothelium/pericyte ratio from approximately 1:1 to 4:1, leading to BRB disruption in patients with DR [26]. Reportedly, a high-glucose environment induces pericyte damage and even apoptosis, which further leads to impaired TJs between endothelial cells. Currently, the primary treatments for DR include intravitreal injections of anti-vascular endothelial growth factor drugs and retinal photocoagulation, which usually aim to regress the neovascularization instead of normalizing the neovascularization [27–29]. The goal of these treatments is of great clinical significance as normalizing the retinal neovascularization could improve the retinal ischemic condition and reverse DR progression. Our study found that under both physiological and pathological settings (OIR and STZ-induced DR), M2-exo could normalize the endothelial cell-to-pericyte ratio and alleviate vascular leakage.

The present study found that M2 microglia were involved in physiological angiogenesis, as revealed by immunofluorescence staining of the mouse retinas at P0, P4, P7, and P14. In OIR mice, treatment with M2-exo promoted normalized blood vessel growth and accelerated the recovery of avascular areas in the retinas. Furthermore, treatment of STZ-induced DR mice with M2-exo showed that they inhibited pericyte apoptosis and promoted endothelial cell proliferation, thereby normalizing the endothelial cell-to-pericyte ratio and reducing vascular leakage. In vitro experiments revealed that M2-exo inhibited pericyte apoptosis as well as promoted the proliferation, tube formation, and migration of endothelial cells under high-glucose conditions. Pericytes were then co-cultured with endothelial cells to mimic blood vessels, and the findings validated our hypothesis that M2-exo could reduce vascular barrier leakage, as evidenced by TEER and FITC-dextran assays. Of note, flow cytometry analysis conducted to analyze M2-exo-treated OIR retinas revealed that M2-exo could polarize microglia towards the M2 type. This further amplified the protective effect of M2 microglia on the retinal vasculature.

M2 microglia-derived exosomes have been indicated to promote the recovery of the crural cord following injury in mice [12]. Moreover, M2 microglia exosomes can promote endothelial cell proliferation and thus glioblastoma haematopoiesis [30]. It has been well documented that the activation of M2 microglia results in overexpression of genes involved in reducing inflammation as well as regulating the immune system, homeostasis, clearance, angiogenesis, and wound healing [31–33]. Ronaldson et al. [34] have shown that M2 microglia protect the blood–brain barrier and reduce leakage by promoting pericyte and endothelial cell homeostasis [34]. Physiologically, the retinal microvasculature consists of pericytes and endothelial cells. In the current study, M2-exo reduced vascular leakage by alleviating pericyte apoptosis and promoting vascular endothelial cell proliferation, thereby regulating the endothelial cell-to-pericyte ratio and protecting the vascular barrier. A few previous studies have shown that DR continues to progress after long-term hyperglycemic stimulation, even if the blood glucose is later controlled, a process known as metabolic memory [35]. In early diabetic retinas, M2 microglia form the primary microglial cluster, whereas M1 microglia dominate as the disease progresses or in the later DR stages. In the present study, we further demonstrated that M2-exo promoted the polarization of microglia towards the M2 phenotype, which reverses the course of DR and thus treats the vasculopathy of DR.

In conclusion, our study demonstrates M2 polarized microglia are the predominant microglia under both pathological and physiological conditions within the retinal microenvironment. The activation of M2 microglia contributes to the normalization of retinal microvascular and the prevention of vascular leakage. This protective effect of M2 microglia on retinal vascular function is mediated by the secreted exosomes. Thus, the findings of this study shed light on a new strategy to prevent diabetes mellitus-induced vascular complications using an exosome-based approach.

### Supplementary Information

**Additional file 1: Video S1.** The video of hRECs uptake of exosomes labelled by Dil. Yellow arrows indicate uptaken exosomes.

**Additional file 2: Figure S1.** M2-exo promotes STZ-induced DR microglia M2 polarisation in retinal. (A) M2 microglia polarization by immunofluorescence in STZ mouse (scale bar = 100 μm). (B) Retinal cell flow analysis demonstrates that M2-exo promotes M2-type microglial polarization.

**Additional file 3: Table S1.** Proliferative membranes were collected from 15 patients with PDR and 15 matched patients with PVR.

## Data Availability

All data are available from the corresponding authors upon reasonable request.

## Abbreviations

DR: Diabetic retinopathy
OIR: Oxygen-induced retinopathy
M2-exo: Exosomes derived from M2 polarized microglia
M0-exo: Exosomes derived from M0 polarized microglia
ECs: Endothelial cells
STZ: Streptozotocin
PDR: Proliferative diabetic retinopathy
PVR: Proliferative vitreoretinopathy
hRECs: Human primary endothelial cells
TEM: Transmission electron microscopy
NTA: Nanoparticle tracking analysis
PFA: Paraformaldehyde
BRB: Blood–retinal barrier
PAS: Periodic acid-Schiff hematoxylin
