# Supplementary figures and images for: M2 microglia-derived exosomes promote vascular remodeling in diabetic retinopathy
Source: J Nanobiotechnology. 2024 Feb 9;22:56. doi: 10.1186/s12951-024-02330-w (PMC10854107; doi:10.1186/s12951-024-02330-w)

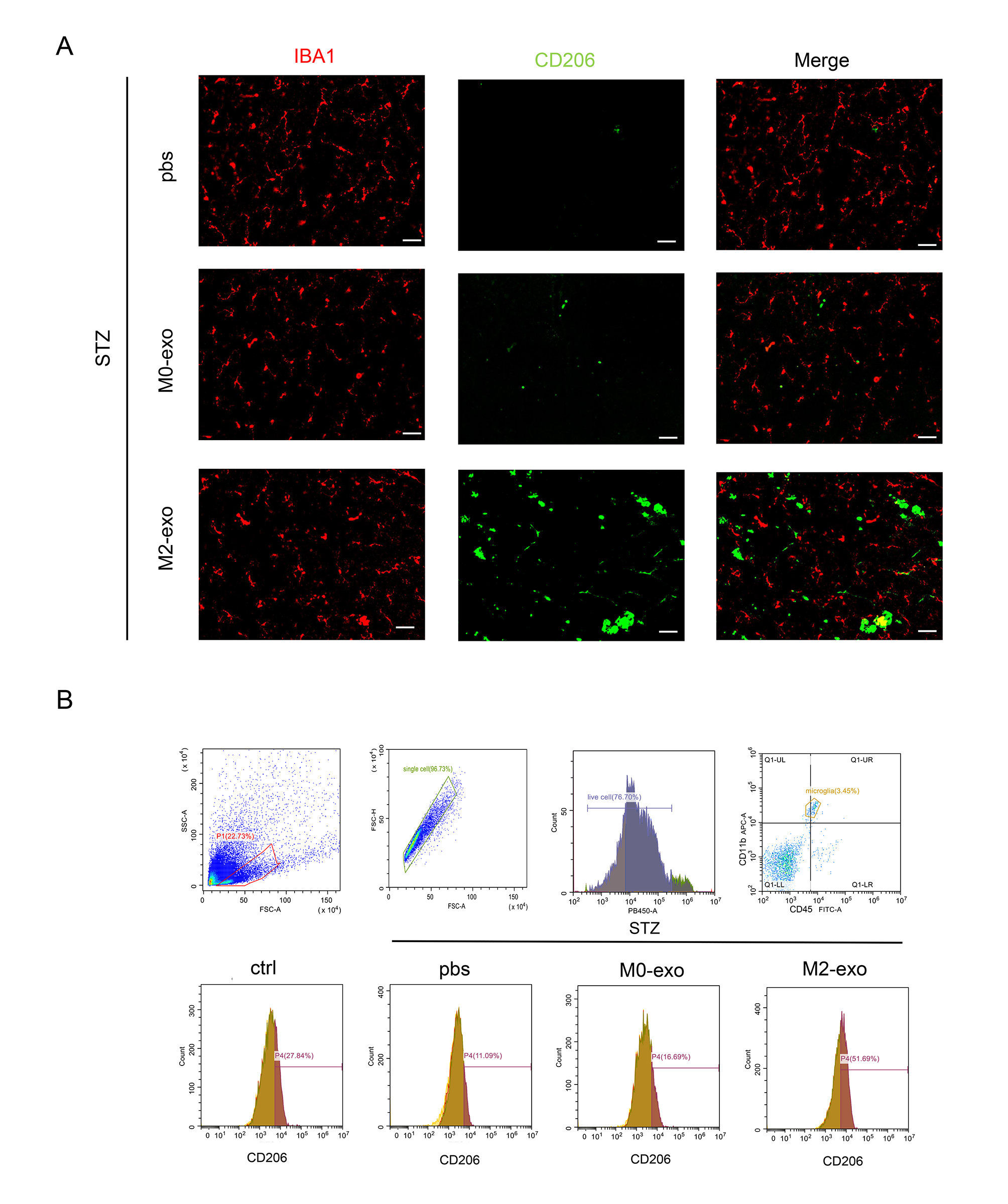

Supplement: Supplementary file 2 — Additional file 2: Figure S1. M2-exo promotes STZ-induced DR microglia M2 polarisation in retinal. (A) M2 microglia polarization by immunofluorescence in STZ mouse (scale bar = 100 μm). (B) Retinal cell flow analysis demonstrates that M2-exo promotes M2-type microglial polarization. [file 12951_2024_2330_MOESM2_ESM.tif]
