# Supplementary material for: M2 microglia-derived exosomes promote vascular remodeling in diabetic retinopathy
Source: J Nanobiotechnology. 2024 Feb 9;22:56. doi: 10.1186/s12951-024-02330-w (PMC10854107; doi:10.1186/s12951-024-02330-w)
Supplement: Supplementary file 3 — Additional file 3: Table S1. Proliferative membranes were collected from 15 patients with PDR and 15 matched patients with PVR. [file 12951_2024_2330_MOESM3_ESM.pdf]

Supplementary Table S1

Proliferative membranes were collected from 15 patients with PDR and 15 matched patients with PVR

Patient	Sex	Age(years) (P=0.7264)	Sample
1	Male	54	PDR1
2	Female	63	PDR2
3	Male	74	PDR3
4	Female	59	PDR4
5	Male	40	PDR5
6	Male	28	PDR6
7	Female	53	PDR7
8	Male	62	PDR8
9	Male	59	PDR9
10	Female	61	PDR10
11	Male	59	PDR11
12	Female	68	PDR12
13	Female	54	PDR13
14	Female	49	PDR14
15	Female	52	PDR15
1	Male	54	PVR1
2	Female	59	PVR2
3	Female	70	PVR3
4	Male	68	PVR4
5	Female	71	PVR5
6	Male	72	PVR6
7	Female	67	PVR7
8	Male	62	PVR8
9	Male	65	PVR9
10	Male	60	PVR10
11	Male	45	PVR11
12	Female	75	PVR12
13	Female	64	PVR13
14	Female	67	PVR14
15	Female	62	PVR15

Sex was not considered a factor in the statistical analysis of the data.
